# Reconstruction of the Femoral Diaphysis Using the Vascularized Fibula Flap: A Case Report

**DOI:** 10.7759/cureus.82000

**Published:** 2025-04-10

**Authors:** Ketevan Kuzanov, Emma K Bremberg, Malemnganbi Soram, Aleksandre Kuzanov, Ivane Kuzanov

**Affiliations:** 1 Medicine, Riga Stradins University, Riga, LVA; 2 Plastic and Reconstructive Surgery, Kuzanov Clinic, Tbilisi, GEO

**Keywords:** external osteosynthesis, femoral diaphysis defect, microsurgery, osteomyelitis, plastic and reconstructive surgery, vascularized fibula flap

## Abstract

The vascularized fibula flap is often utilized in various reconstructive surgeries to cover a bone or soft tissue defect due to its generous length, adequate vascularization, and the possibility of removal with minimal loss of limb function. We present a 32-year-old male patient with a 27-cm femoral diaphysis defect caused by a high-impact car accident, which led to multiple femoral fractures and was further complicated by osteomyelitis. Initial consultations with other physicians advised limb amputation as the sole approach due to the severity of the case and potential complications. Upon presentation at Kuzanov Clinic, the possibility of salvaging the limb using the vascularized fibula flap was proposed. A 27-cm fibula flap was harvested and transplanted to the femoral defect. Anastomosis was established between the vasculature of the flap and the recipient zone. A few months after the surgery, an upper fibular fracture occurred, which later healed, and the fibula hypertrophied. At a 10-year follow-up, the patient remains ambulatory, with limb shortening effectively managed with a custom shoe.

## Introduction

Salvage of the limb poses a challenge, as the optimal technique remains debated among experts, with no consensus reached [1]. When attempting such surgeries, it is crucial to consider neighboring vital structures [2], such as vascularity, innervation, surrounding tissues, and skeletal integrity [3].

The vascularized fibula flap was first reportedly used in 1975 to reconstruct a tibial defect [4] and has since been commonly used for the reconstruction of jaw defects following trauma, tumor resection, congenital deformities, and osteomyelitis [5]. The vascularized fibula flap is particularly favored among surgeons due to its generous length, proper vascularization, and the possibility of removal with minimal loss of limb function, given the tibia’s ability to bear the weight alone [5]. In this case report, we will discuss such a flap covering the defect of the femoral diaphysis.

## Case presentation

**Figure 1:**
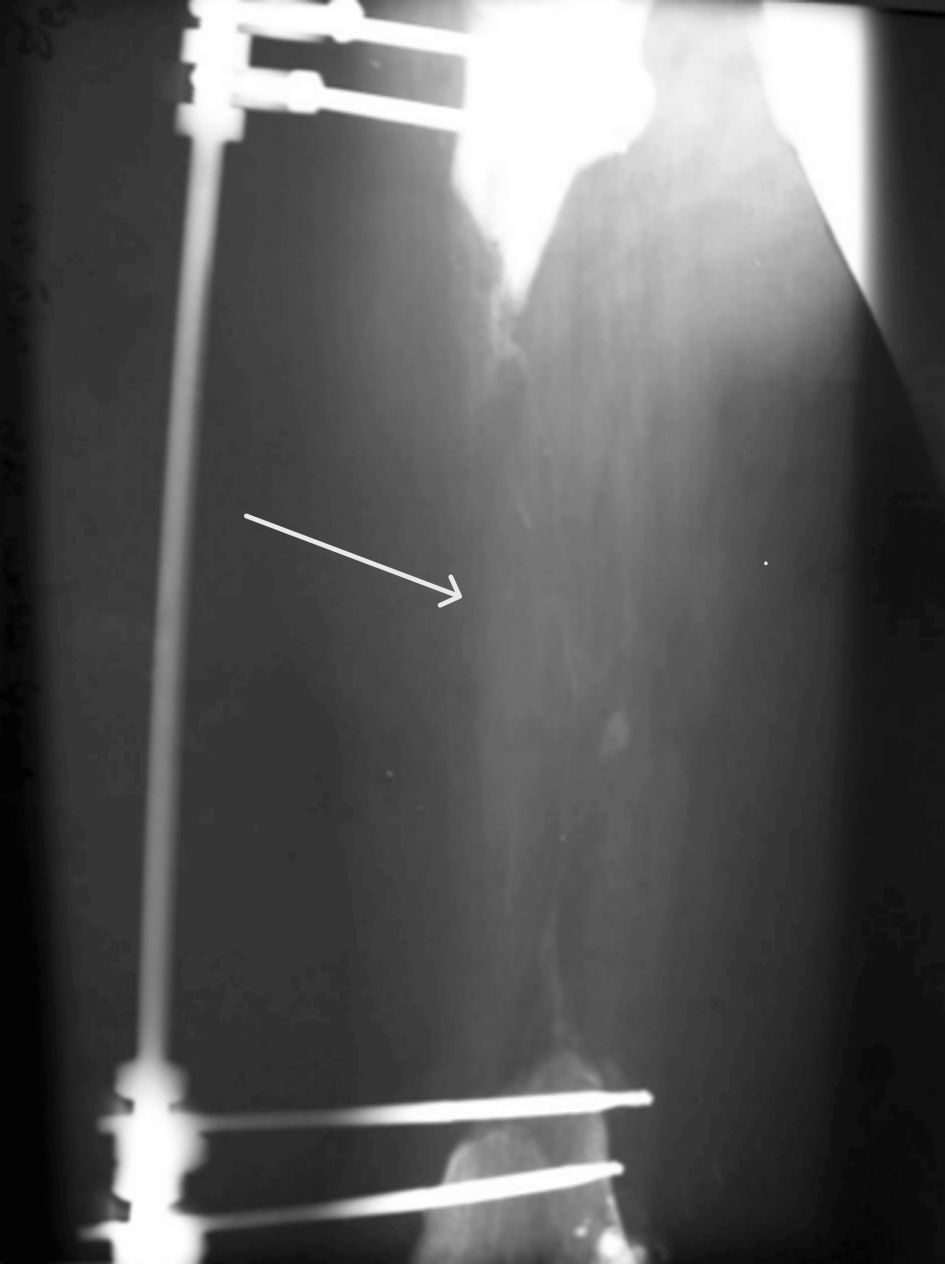
X-ray image depicting femoral diaphysis defect following the removal of diaphyseal fragments. Missing femoral diaphysis is indicated by the arrow.

A 32-year-old man sustained multiple bone fractures including a comminuted femoral fracture following a traumatic car accident. His condition was further complicated by osteomyelitis, necessitating a revision surgery to remove the fragmented diaphysis. As a result, only the epiphyseal ends of the femur were left behind (Figure 1). Total limb amputation was considered the sole viable option.

**Figure 2:**
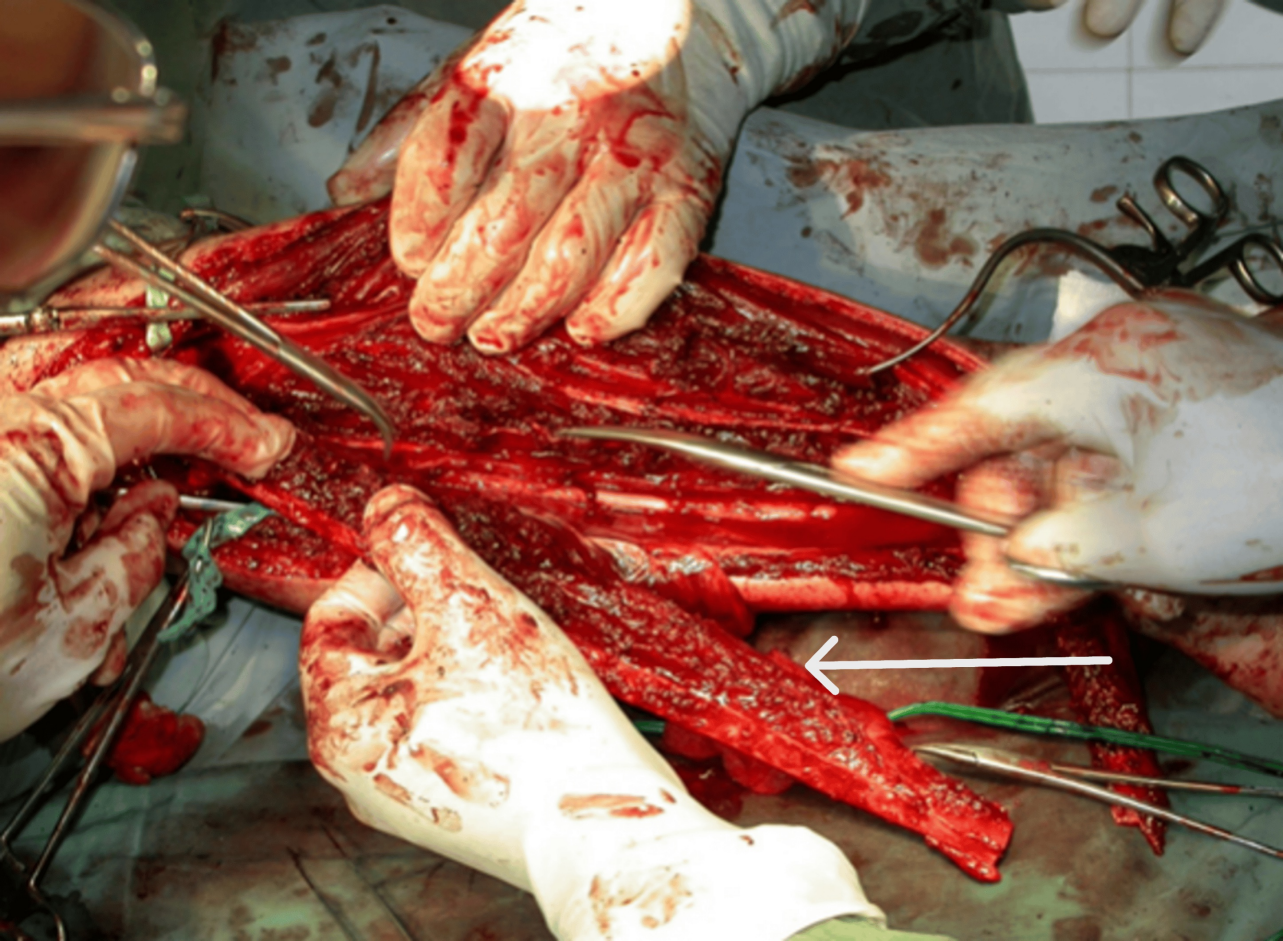
Removal of the vascularized fibula. The vascularized fibula flap is indicated by the arrow.

**Figure 3:**
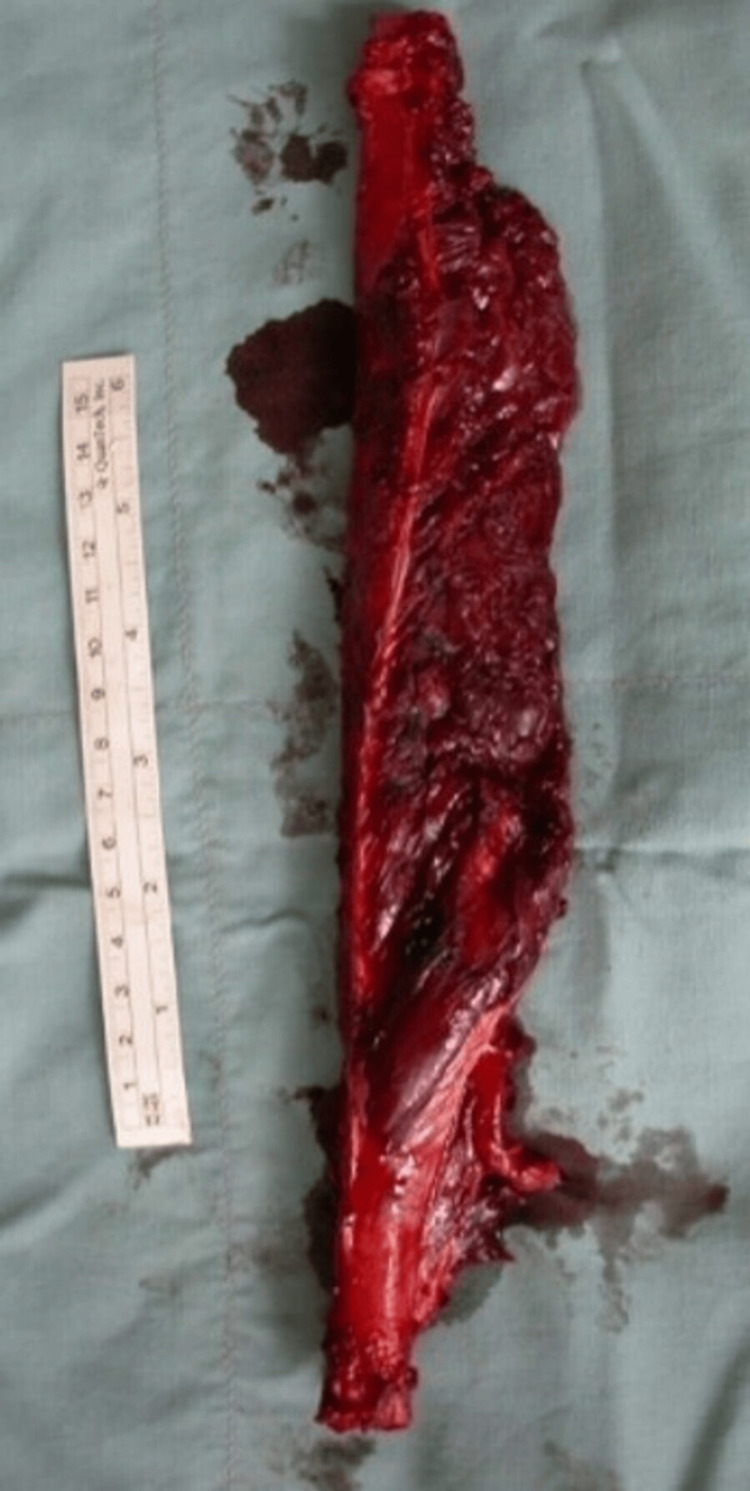
The vascularized fibula flap.

**Figure 4:**
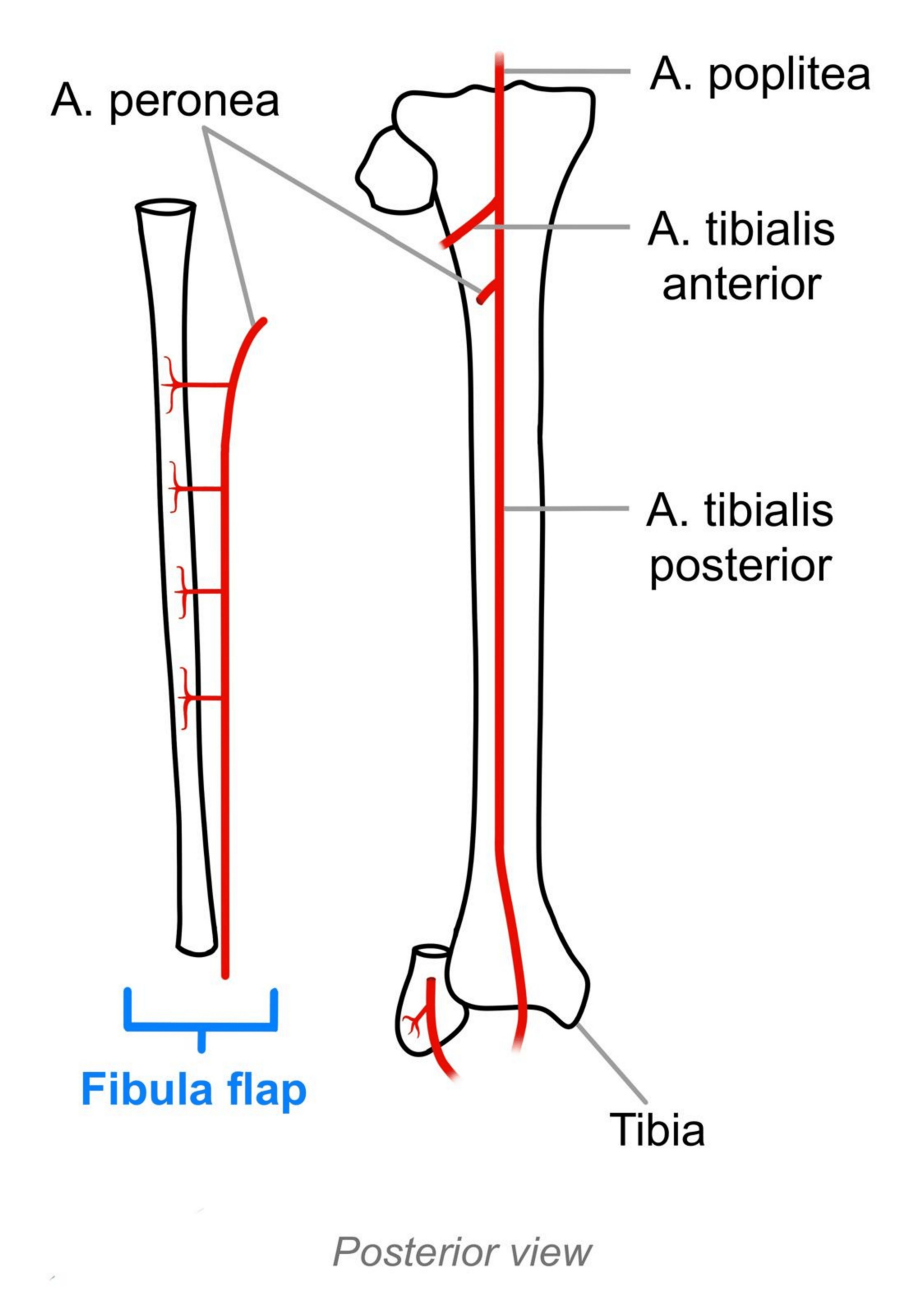
Anatomical visual of the vascularized fibula flap. Creation and property of Emma K. Bremberg

Upon his presentation at Kuzanov Clinic, the femoral defect measured 27 cm. The possibility of salvaging the limb was proposed by the method of transplanting the vascularized fibula flap to the femoral defect. A 27-cm fibula was harvested from the opposite leg, along with the peroneal vessels, and transplanted to the diaphyseal defect (Figures 2-4).

**Figure 5:**
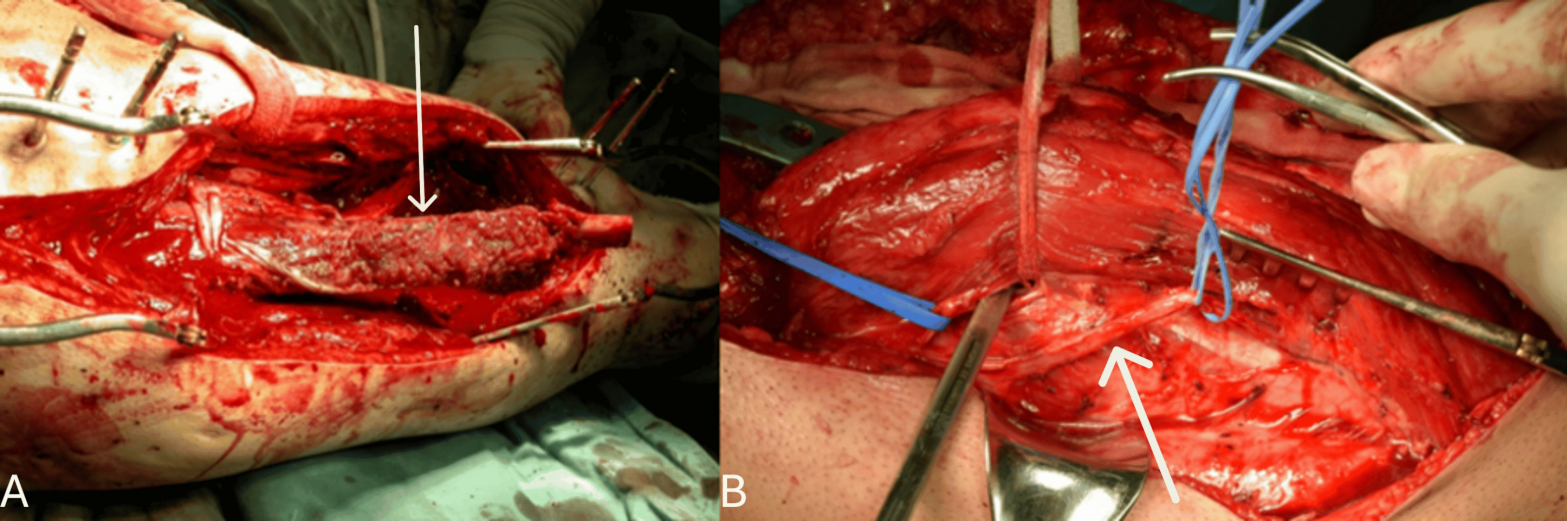
Creation of anastomosis between the vasculature of the flap and the recipient zone. Inserting the vascularized fibula flap (as indicated by the arrow) into the recipient zone in (A). Positioning the descending branch of the lateral circumflex femoral artery (as indicated by the arrow) beneath the rectus femoris muscle to facilitate its connection to the flap pedicle in (B).

An end-to-end anastomosis was created between the vascular pedicle of the flap and the descending branch of the lateral circumflex femoral artery (Figure 5). This artery was chosen, since all the major arteries were located too deep and anastomosis with them would require a vein extension graft. Vein graft adaptation to arterial circulation poses an increased risk of thrombosis due to venous endothelial injury caused by the comparably higher pressure in arterial circulation [6]. To ensure that the descending branch of the lateral circumflex femoral artery reaches the flap pedicle, it was positioned beneath the rectus femoris muscle (Figure 5).

**Figure 6:**
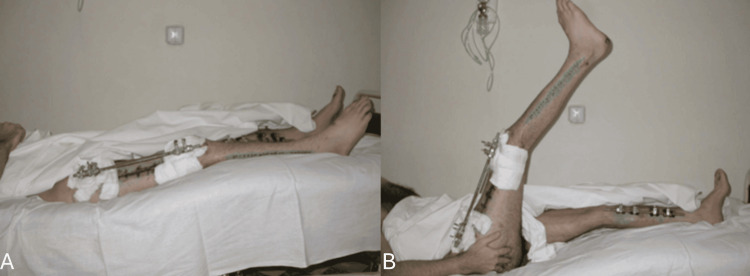
Ten days postsurgery. The patient is demonstrating early mobility against gravity. An external fixation device was used for osteosynthesis. The patient lifts his leg (B) from a resting position (A).

Doppler ultrasonography was used to evaluate the perfusion of the recipient site, which was satisfactory. An external fixation device was used for osteosynthesis (Figure 6).

**Figure 7:**
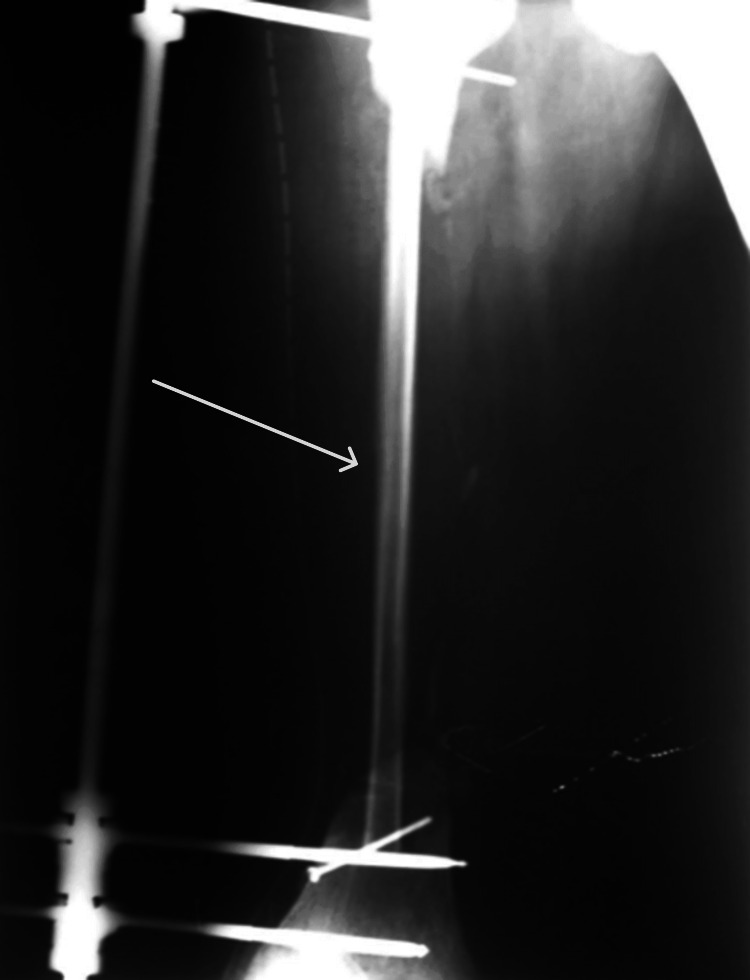
X-ray image taken 2 weeks postsurgery showing a thin fibula. The thin fibula is indicated by the arrow.

Two weeks after the surgery, the transplanted fibula appeared thin on X-ray (Figure 7).

**Figure 8:**
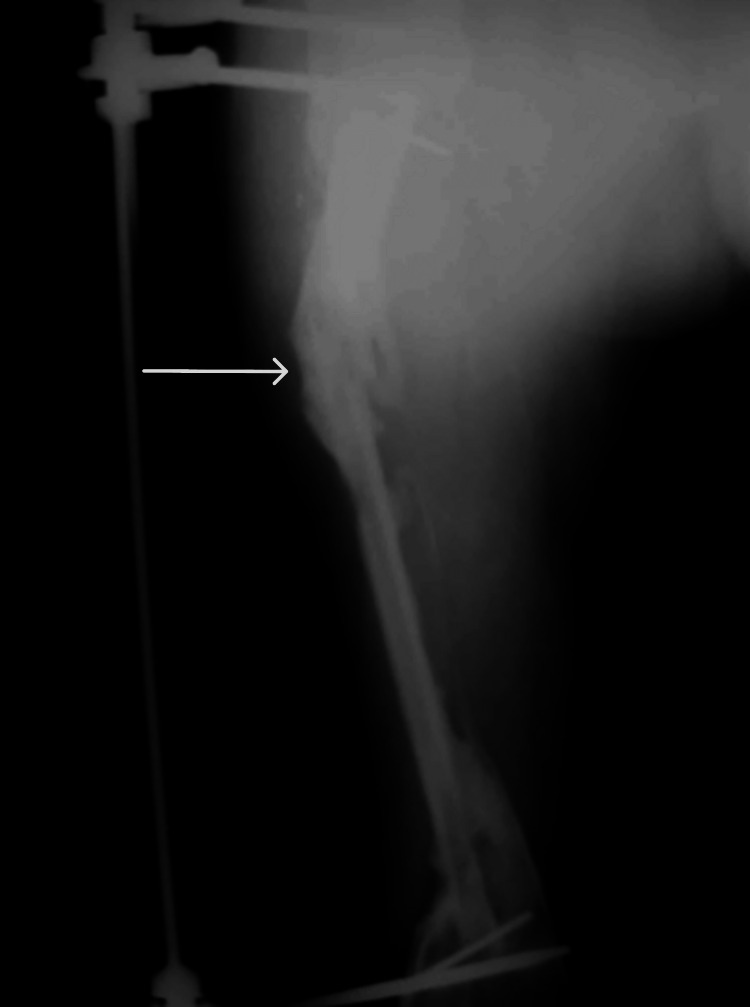
X-ray image 4 months postsurgery showing an upper fibular fracture. Upper fibular fracture is indicated by the arrow.

Four months later, the transplanted fibula sustained an upper fracture, which healed, followed by thickening of the flap (Figure 8).

**Figure 9:**
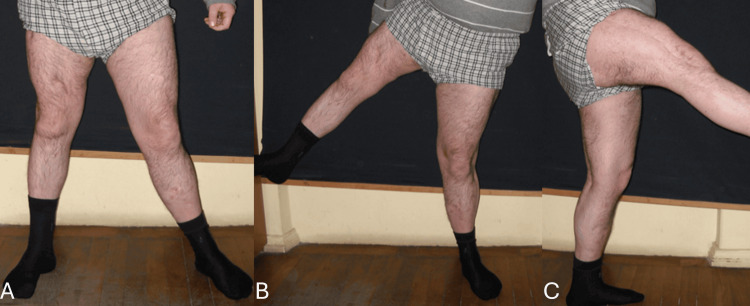
A 10-year follow-up demonstrating the patient remains ambulatory. The patient is demonstrating the weight-bearing ability of the transplanted leg in (A) and its range of motion in (B and C).

A 10-year follow-up confirmed that no further fractures had occurred and the patient remained ambulatory (Figure 9). The length of the operated leg was shortened due to improper healing. This was efficiently corrected by using a customized shoe with a heel. In order to prevent further fractures, a cane was recommended to relieve the weight from the transplanted fibula. Additionally, the patient expressed satisfaction with the overall outcome.

## Discussion

Limb amputation not only results in the physical loss of a body part but also imposes emotional and social burdens. Many amputees experience hopelessness, depression, and body image distress [7], which is why it is crucial to explore all possible limb-salvaging options and consider amputation as the last resort.

With advances in artificial limb technology, some may argue that a bionic prosthesis would have been a more practical alternative. However, it is important to consider the patient's financial capabilities, the availability of such a product in their region, and, most importantly, their desire to preserve the limb.

A primary goal of limb salvage is to facilitate the patient’s return to normal life [8]. However, postoperative patient education is essential regarding weight-bearing limitations and fracture risks associated with non-compliance. Proper use of an assistive mobility device such as a cane ensures adequate weight distribution [9], thereby reducing the excessive load of the transplanted fibula.

This surgical approach is more suited for patients who lack access to high-end bionic prosthetics or who may struggle with frequent check-ups for their artificial limbs. As mentioned before, patient autonomy should be a primary consideration in decision-making; thus, the main indication for such limb salvage surgery would be the patient's decision to preserve their limb.

## Conclusions

This case report demonstrates successful limb salvage using a vascularized fibula flap to cover a femoral defect. This flap not only serves as a vital source of bone graft but also offers coverage for soft tissue, making it a highly versatile option. When performed with the proper surgical technique, it can help restore both the structural strength and functionality of the femoral diaphysis. Despite the postoperative fracture and limb shortening, the patient remains ambulatory, and the mismatch of the limb length is corrected with a customized shoe. The surgery has proven to be a long-term success, as demonstrated by the patient's satisfaction with the outcome and his fully functional mobility 10 years postoperatively.

## References

[1] Migliorini F, La Padula G, Torsiello E, Spiezia F, Oliva F, Maffulli N (2021). Strategies for large bone defect reconstruction after trauma, infections or tumour excision: a comprehensive review of the literature. Eur J Med Res.

[2] Kadam D (2013). Limb salvage surgery. Indian J Plast Surg.

[3] Beeharry MW, Walden-Smith T, Moqeem K (2022). Limb salvage vs. amputation: factors influencing the decision-making process and outcomes for mangled extremity injuries. Cureus.

[4] Taylor GI, Miller GD, Ham FJ (1975). The free vascularized bone graft. A clinical extension of microvascular techniques. Plast Reconstr Surg.

[5] Taqi M, Hohman MH, Raju S (2025). Fibula free flaps. StatPearls [Internet].

[6] Muto A, Model L, Ziegler K, Eghbalieh SD, Dardik A (2010). Mechanisms of vein graft adaptation to the arterial circulation: insights into the neointimal algorithm and management strategies. Circ J.

[7] Abouammoh N, Aldebeya W, Abuzaid R (2021). Experiences and needs of patients with lower limb amputation in Saudi Arabia: a qualitative study. East Mediterr Health J.

[8] (2025). Limb salvage. https://www.pennmedicine.org/departments-and-centers/orthopaedic-surgery/about-us/excellence-in-motion-newsletter/archive/2018-newletter/limb-salvage.

[9] Sehgal M, Jacobs J, Biggs WS (2021). Mobility assistive device use in older adults. Am Fam Physician.

